# Tetra­kis(di-4-pyridylsulfane)dinitratocopper(II)

**DOI:** 10.1107/S1600536810011414

**Published:** 2010-04-02

**Authors:** Rong-Hua Gao, Li-Li Yang

**Affiliations:** a Weihai Department of Biological and Chemical Engineering, Weihai Vocational College, Weihai 264210, People’s Republic of China

## Abstract

In the title complex, [Cu(NO_3_)_2_(C_10_H_8_N_2_S)_4_], the Cu^II^ atom (site symmetry 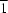) is coordinated by two monodentate nitrate ions and two monodentate di-4-pyridylsulfane ligands, resulting in a slightly distorted *trans*-arranged CuO_2_N_4_ octa­hedral geometry. Intra­molecular C—H⋯O hydrogen bonds are present. In the crystal, adjacent mol­ecules are linked *via* C—H⋯N hydrogen bonds into chains parallel to the *a* axis. Inter­molecular C—H⋯O inter­actions also occur.

## Related literature

For transition-metal complexes of di-4-pyridylsulfane, see: Wen *et al.* (2004 ▶); Muthu *et al.* (2005 ▶); Xu *et al.* (2007 ▶); Zhang *et al.* (2008 ▶).
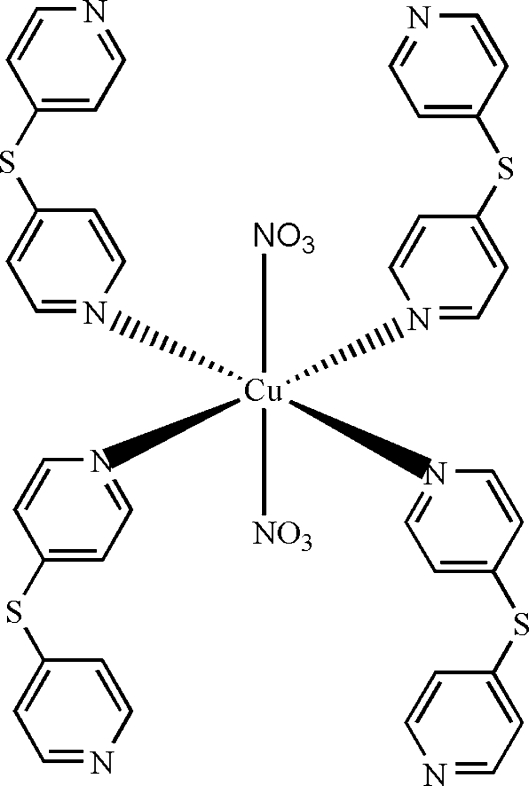


## Experimental

### 

#### Crystal data

[Cu(NO_3_)_2_(C_10_H_8_N_2_S)_4_]
*M* *_r_* = 940.59
Triclinic, 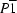

*a* = 9.299 (4) Å
*b* = 10.765 (5) Å
*c* = 10.978 (5) Å
α = 84.408 (6)°
β = 73.759 (6)°
γ = 79.180 (6)°
*V* = 1035.1 (8) Å^3^
*Z* = 1
Mo *K*α radiation
μ = 0.79 mm^−1^
*T* = 296 K
0.21 × 0.19 × 0.17 mm

#### Data collection

Bruker SMART APEXII diffractometer
Absorption correction: multi-scan (*SADABS*; Bruker, 2000 ▶) *T*_min_ = 0.847, *T*_max_ = 0.874
7479 measured reflections
3728 independent reflections
2392 reflections with *I* > 2σ(*I*)
*R*_int_ = 0.061

#### Refinement

*R*[*F* ^2^ > 2σ(*F* ^2^)] = 0.044
*wR*(*F* ^2^) = 0.100
*S* = 0.90
3679 reflections
277 parameters
6 restraints
H-atom parameters constrained
Δρ_max_ = 0.43 e Å^−3^
Δρ_min_ = −0.33 e Å^−3^

### 

Data collection: *APEX2* (Bruker, 2004 ▶); cell refinement: *SAINT* (Bruker, 2004 ▶); data reduction: *SAINT*; program(s) used to solve structure: *SHELXS97* (Sheldrick, 2008 ▶); program(s) used to refine structure: *SHELXL97* (Sheldrick, 2008 ▶); molecular graphics: *ORTEP-3 for Windows* (Farrugia, 1997 ▶) and *DIAMOND* (Brandenburg, 2006 ▶); software used to prepare material for publication: *SHELXL97* and *PLATON* (Spek, 2009 ▶).

## Supplementary Material

Crystal structure: contains datablocks I, global. DOI: 10.1107/S1600536810011414/rz2428sup1.cif

Structure factors: contains datablocks I. DOI: 10.1107/S1600536810011414/rz2428Isup2.hkl

Additional supplementary materials:  crystallographic information; 3D view; checkCIF report

## Figures and Tables

**Table 1:** Hydrogen-bond geometry (Å, °)

*D*—H⋯*A*	*D*—H	H⋯*A*	*D*⋯*A*	*D*—H⋯*A*
C11—H11⋯O1^i^	0.93	2.52	3.063 (4)	118
C5—H5⋯O2^i^	0.93	2.49	3.419 (4)	174
C5—H5⋯O1^i^	0.93	2.51	3.193 (4)	130
C14—H14⋯N4^ii^	0.93	2.47	3.279 (5)	146
C1—H1⋯O1	0.93	2.27	3.008 (4)	135

Symmetry codes: (i) 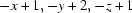; (ii) 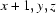.

**Fig. 1.**
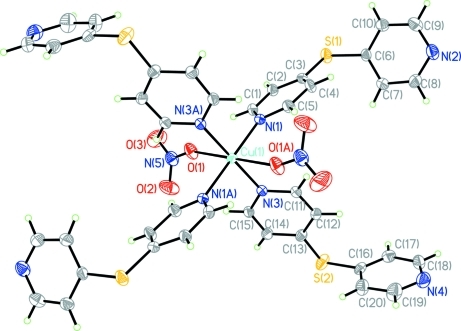
The molecular structure of the title compound with atomic labeling scheme and displacement ellipsoids drawn at the 50% probability level [symmetry code: (A) -x+1,-y+2,-z+1].

**Fig. 2.**
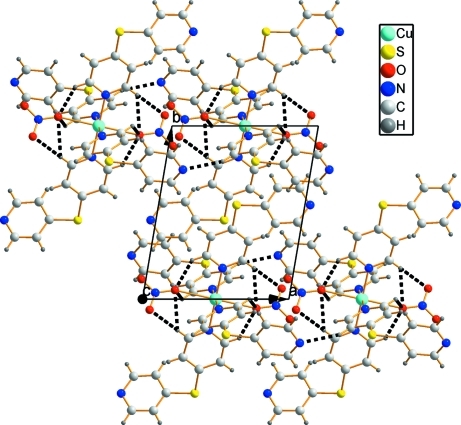
Crystal packing of the title compound viewed along the c axis. Hydrogen bonds are drawn as dashed lines.

## supplementary crystallographic information

### Comment

Flexible ligands are interesting due to their smaller steric effects, which contribute to the construction of novel complexes. Compared to the widely investigated 4,4'-bipy, the dps (di-4-pyridylsulfane) ligand is more flexible and the two pyridine rings can rotate freely. There are only few complexes of metal-organic compounds with dps (Xu *et al.*, 2007; Wen *et al.*, 2004; Muthu *et al.*, 2005; Zhang *et al.*, 2008). Herein, we report the synthesis and structure of the title compound, dinitratotetrakis(di-4-pyridylsulfane)copper(II).

The title compound (Fig. 1) crystallizes in the monoclinic space group P1. The copper(II) ion lies on a crystallography inversion centre and adopts a slightly distorted octahedral provided by four N atoms from two dps ligands in the equatorial plane and two O atoms from two nitrate ions in the axial position. In the equatorial plane, the Cu1—N1 and Cu1—N3 bond lengths are 2.047 (3) and 2.023 (3) Å respectively, while the Cu1—O1 axial bond length is 2.558 (3) Å. The conformation of the complex molecule is stabilized by intramolecular C—H···O hydrogen bonds (Table 1). In the crystal structure (Fig. 2), intermolecular C—H···N hydrogen interactions link molecules into chains parallel to the *a* axis.

### Experimental

The title compound was prepared by adding a solution of copper(II) nitrate hexahydrate (0.1 mmol) in water (6 ml) to a solution of di-4-pyridylsulfane (0.2 mmol) in CH_3_OH (5 ml) with gentle stirring. After several days, block-shaped blue crystals suitable for X-ray analysis were obtained on slow evaporation of the solvent (Yield 30 mg; 31.9%, based on Cu). Anal. calcd for C_40_H_32_CuN_10_O_6_S_4_ (940.59): C 51.08, H 3.43, N 14.89%; found: C 51.23, H 3.45, N 14.93%.

### Refinement

All H atoms were positioned geometrically and refined as riding, with C—H = 0.93 Å, and with *U*_iso_ (H) = 1.2*U*_eq_(C). The anisotropic displacement parameters of atoms O1 and N5 were restrained to be similar (i.e. the SIMU restraint was applied).

### Crystal data

**Table:**

[Cu(NO_3_)_2_(C_10_H_8_N_2_S)_4_]	*Z* = 1
*M**_r_* = 940.59	*F*(000) = 483
Triclinic, *P*1	*D*_x_ = 1.509 Mg m^−^^3^
Hall symbol: -P 1	Mo *K*α radiation, λ = 0.71073 Å
*a* = 9.299 (4) Å	Cell parameters from 4796 reflections
*b* = 10.765 (5) Å	θ = 2.3–28.2°
*c* = 10.978 (5) Å	µ = 0.79 mm^−^^1^
α = 84.408 (6)°	*T* = 296 K
β = 73.759 (6)°	Block, blue
γ = 79.180 (6)°	0.21 × 0.19 × 0.17 mm
*V* = 1035.1 (8) Å^3^	

### Data collection

**Table:**

Bruker SMART APEXII diffractometer	3728 independent reflections
Radiation source: fine-focus sealed tube	2392 reflections with *I* > 2σ(*I*)
graphite	*R*_int_ = 0.061
ω scans	θ_max_ = 25.2°, θ_min_ = 1.9°
Absorption correction: multi-scan (*SADABS*; Bruker, 2000)	*h* = −11→11
*T*_min_ = 0.847, *T*_max_ = 0.874	*k* = −12→12
7479 measured reflections	*l* = −13→12

### Refinement

**Table:**

Refinement on *F*^2^	Primary atom site location: structure-invariant direct methods
Least-squares matrix: full	Secondary atom site location: difference Fourier map
*R*[*F*^2^ > 2σ(*F*^2^)] = 0.044	Hydrogen site location: inferred from neighbouring sites
*wR*(*F*^2^) = 0.100	H-atom parameters constrained
*S* = 0.90	*w* = 1/[σ^2^(*F*_o_^2^) + (0.0391*P*)^2^] where *P* = (*F*_o_^2^ + 2*F*_c_^2^)/3
3679 reflections	(Δ/σ)_max_ < 0.001
277 parameters	Δρ_max_ = 0.43 e Å^−^^3^
6 restraints	Δρ_min_ = −0.33 e Å^−^^3^

### Special details

**Table:**

Geometry. All e.s.d.'s (except the e.s.d. in the dihedral angle between two l.s. planes) are estimated using the full covariance matrix. The cell e.s.d.'s are taken into account individually in the estimation of e.s.d.'s in distances, angles and torsion angles; correlations between e.s.d.'s in cell parameters are only used when they are defined by crystal symmetry. An approximate (isotropic) treatment of cell e.s.d.'s is used for estimating e.s.d.'s involving l.s. planes.
Refinement. Refinement of *F*^2^ against ALL reflections. The weighted *R*-factor wR and goodness of fit *S* are based on *F*^2^, conventional *R*-factors *R* are based on *F*, with *F* set to zero for negative *F*^2^. The threshold expression of *F*^2^ > σ(*F*^2^) is used only for calculating *R*-factors(gt) etc. and is not relevant to the choice of reflections for refinement. *R*-factors based on *F*^2^ are statistically about twice as large as those based on *F*, and *R*- factors based on ALL data will be even larger.

### Fractional atomic coordinates and isotropic or equivalent isotropic displacement parameters (Å^2^)

**Table:**

	*x*	*y*	*z*	*U*_iso_*/*U*_eq_	
Cu1	0.5000	1.0000	0.5000	0.03494 (19)	
S1	0.46635 (10)	0.45645 (8)	0.29541 (10)	0.0526 (3)	
S2	0.38860 (11)	1.20522 (11)	−0.05513 (9)	0.0609 (3)	
O1	0.7889 (3)	0.9473 (2)	0.4138 (2)	0.0586 (7)	
O2	0.9388 (3)	1.0613 (3)	0.2949 (3)	0.0869 (10)	
O3	1.0298 (3)	0.8845 (3)	0.3704 (3)	0.1064 (12)	
N1	0.4896 (3)	0.8203 (2)	0.4611 (2)	0.0337 (6)	
N2	−0.0360 (3)	0.4563 (3)	0.3369 (3)	0.0556 (9)	
N3	0.4666 (3)	1.0575 (2)	0.3276 (2)	0.0327 (6)	
N4	−0.1253 (4)	1.2525 (3)	0.0096 (3)	0.0642 (9)	
N5	0.9202 (4)	0.9628 (3)	0.3612 (3)	0.0538 (7)	
C1	0.6125 (4)	0.7481 (3)	0.3889 (3)	0.0381 (8)	
H1	0.7061	0.7748	0.3711	0.046*	
C2	0.6058 (4)	0.6377 (3)	0.3409 (3)	0.0381 (8)	
H2	0.6937	0.5908	0.2917	0.046*	
C3	0.4684 (4)	0.5955 (3)	0.3654 (3)	0.0359 (8)	
C4	0.3437 (4)	0.6656 (3)	0.4452 (3)	0.0433 (9)	
H4	0.2500	0.6385	0.4682	0.052*	
C5	0.3596 (4)	0.7753 (3)	0.4899 (3)	0.0413 (9)	
H5	0.2746	0.8210	0.5436	0.050*	
C6	0.2705 (3)	0.4599 (3)	0.3124 (3)	0.0385 (8)	
C7	0.1802 (4)	0.5620 (3)	0.2676 (4)	0.0510 (10)	
H7	0.2205	0.6326	0.2270	0.061*	
C8	0.0298 (4)	0.5550 (4)	0.2851 (4)	0.0560 (10)	
H8	−0.0309	0.6253	0.2586	0.067*	
C9	0.0547 (4)	0.3585 (3)	0.3756 (4)	0.0556 (10)	
H9	0.0133	0.2866	0.4105	0.067*	
C10	0.2053 (4)	0.3570 (3)	0.3674 (3)	0.0453 (9)	
H10	0.2621	0.2871	0.3987	0.054*	
C11	0.3401 (4)	1.0425 (3)	0.2976 (3)	0.0365 (8)	
H11	0.2705	1.0005	0.3569	0.044*	
C12	0.3082 (4)	1.0855 (3)	0.1852 (3)	0.0386 (8)	
H12	0.2191	1.0726	0.1693	0.046*	
C13	0.4103 (4)	1.1485 (3)	0.0953 (3)	0.0389 (8)	
C14	0.5424 (4)	1.1637 (3)	0.1250 (3)	0.0404 (9)	
H14	0.6140	1.2051	0.0671	0.049*	
C15	0.5658 (4)	1.1175 (3)	0.2396 (3)	0.0369 (8)	
H15	0.6547	1.1281	0.2575	0.044*	
C16	0.1889 (4)	1.2224 (3)	−0.0291 (3)	0.0415 (9)	
C17	0.1259 (4)	1.1460 (3)	−0.0847 (3)	0.0504 (10)	
H17	0.1873	1.0824	−0.1364	0.061*	
C18	−0.0290 (5)	1.1645 (4)	−0.0632 (4)	0.0592 (11)	
H18	−0.0694	1.1118	−0.1022	0.071*	
C19	0.0927 (4)	1.3150 (4)	0.0459 (4)	0.0603 (11)	
H19	0.1307	1.3701	0.0843	0.072*	
C20	−0.0611 (5)	1.3244 (4)	0.0630 (4)	0.0725 (13)	
H20	−0.1250	1.3859	0.1161	0.087*	

### Atomic displacement parameters (Å^2^)

**Table:**

	*U*^11^	*U*^22^	*U*^33^	*U*^12^	*U*^13^	*U*^23^
Cu1	0.0427 (4)	0.0243 (3)	0.0432 (4)	−0.0055 (2)	−0.0207 (3)	0.0000 (3)
S1	0.0416 (6)	0.0364 (5)	0.0856 (8)	−0.0001 (4)	−0.0239 (5)	−0.0226 (5)
S2	0.0406 (6)	0.0945 (8)	0.0442 (6)	−0.0093 (5)	−0.0137 (5)	0.0163 (6)
O1	0.0421 (14)	0.0601 (14)	0.0723 (17)	−0.0193 (12)	−0.0018 (13)	−0.0171 (13)
O2	0.073 (2)	0.064 (2)	0.107 (3)	−0.0221 (16)	0.0062 (18)	0.0106 (18)
O3	0.069 (2)	0.089 (2)	0.148 (3)	0.0266 (18)	−0.036 (2)	0.000 (2)
N1	0.0358 (16)	0.0265 (14)	0.0415 (17)	−0.0050 (12)	−0.0151 (14)	−0.0005 (12)
N2	0.0396 (18)	0.052 (2)	0.076 (2)	−0.0107 (16)	−0.0115 (17)	−0.0103 (18)
N3	0.0353 (16)	0.0287 (14)	0.0370 (17)	−0.0077 (12)	−0.0128 (14)	−0.0015 (12)
N4	0.045 (2)	0.073 (2)	0.072 (3)	−0.0104 (19)	−0.0154 (19)	0.006 (2)
N5	0.0412 (16)	0.0535 (16)	0.0649 (18)	−0.0102 (15)	−0.0065 (15)	−0.0127 (14)
C1	0.0317 (19)	0.0398 (19)	0.046 (2)	−0.0101 (16)	−0.0133 (17)	−0.0003 (17)
C2	0.035 (2)	0.0339 (19)	0.047 (2)	−0.0037 (15)	−0.0122 (17)	−0.0078 (16)
C3	0.040 (2)	0.0269 (17)	0.044 (2)	−0.0049 (15)	−0.0170 (17)	−0.0005 (15)
C4	0.0293 (19)	0.0364 (19)	0.065 (3)	−0.0094 (15)	−0.0092 (18)	−0.0096 (18)
C5	0.037 (2)	0.0347 (19)	0.051 (2)	−0.0030 (16)	−0.0087 (18)	−0.0083 (17)
C6	0.038 (2)	0.0345 (18)	0.048 (2)	−0.0056 (15)	−0.0179 (17)	−0.0093 (16)
C7	0.049 (2)	0.040 (2)	0.069 (3)	−0.0125 (17)	−0.023 (2)	0.0068 (19)
C8	0.050 (2)	0.046 (2)	0.077 (3)	−0.0013 (19)	−0.030 (2)	−0.001 (2)
C9	0.054 (3)	0.044 (2)	0.065 (3)	−0.0131 (19)	−0.006 (2)	−0.004 (2)
C10	0.047 (2)	0.0341 (19)	0.054 (2)	−0.0027 (17)	−0.0130 (19)	−0.0068 (17)
C11	0.0350 (19)	0.0340 (18)	0.042 (2)	−0.0104 (15)	−0.0114 (17)	0.0008 (16)
C12	0.0321 (19)	0.0406 (19)	0.045 (2)	−0.0078 (15)	−0.0133 (17)	0.0028 (17)
C13	0.0323 (19)	0.044 (2)	0.037 (2)	−0.0021 (16)	−0.0084 (17)	−0.0008 (16)
C14	0.034 (2)	0.043 (2)	0.042 (2)	−0.0099 (16)	−0.0081 (17)	0.0042 (17)
C15	0.0321 (19)	0.0322 (18)	0.049 (2)	−0.0054 (15)	−0.0136 (18)	−0.0035 (17)
C16	0.037 (2)	0.050 (2)	0.036 (2)	−0.0015 (17)	−0.0137 (17)	0.0079 (17)
C17	0.053 (2)	0.045 (2)	0.051 (3)	−0.0016 (18)	−0.015 (2)	−0.0030 (19)
C18	0.062 (3)	0.061 (3)	0.068 (3)	−0.024 (2)	−0.032 (2)	0.006 (2)
C19	0.053 (3)	0.068 (3)	0.065 (3)	−0.006 (2)	−0.021 (2)	−0.018 (2)
C20	0.053 (3)	0.079 (3)	0.080 (3)	0.008 (2)	−0.015 (3)	−0.024 (3)

### Geometric parameters (Å, °)

**Table:**

Cu1—N3^i^	2.023 (3)	C4—H4	0.9300
Cu1—N3	2.023 (3)	C5—H5	0.9300
Cu1—N1^i^	2.047 (3)	C6—C10	1.370 (4)
Cu1—N1	2.047 (3)	C6—C7	1.390 (4)
Cu1—O1	2.558 (3)	C7—C8	1.373 (5)
Cu1—O1^i^	2.558 (3)	C7—H7	0.9300
S1—C3	1.754 (3)	C8—H8	0.9300
S1—C6	1.772 (3)	C9—C10	1.375 (5)
S2—C13	1.757 (3)	C9—H9	0.9300
S2—C16	1.775 (3)	C10—H10	0.9300
O1—N5	1.231 (3)	C11—C12	1.366 (4)
O2—N5	1.238 (4)	C11—H11	0.9300
O3—N5	1.216 (4)	C12—C13	1.387 (4)
N1—C5	1.331 (4)	C12—H12	0.9300
N1—C1	1.348 (4)	C13—C14	1.397 (4)
N2—C8	1.326 (4)	C14—C15	1.367 (4)
N2—C9	1.334 (4)	C14—H14	0.9300
N3—C15	1.344 (4)	C15—H15	0.9300
N3—C11	1.347 (4)	C16—C17	1.367 (4)
N4—C20	1.326 (5)	C16—C19	1.374 (5)
N4—C18	1.329 (5)	C17—C18	1.372 (5)
C1—C2	1.365 (4)	C17—H17	0.9300
C1—H1	0.9300	C18—H18	0.9300
C2—C3	1.383 (4)	C19—C20	1.375 (5)
C2—H2	0.9300	C19—H19	0.9300
C3—C4	1.383 (4)	C20—H20	0.9300
C4—C5	1.370 (4)		
			
N3^i^—Cu1—N3	180.000 (1)	C10—C6—S1	119.4 (3)
N3^i^—Cu1—N1^i^	87.75 (10)	C7—C6—S1	122.4 (3)
N3—Cu1—N1^i^	92.25 (10)	C8—C7—C6	117.8 (3)
N3^i^—Cu1—N1	92.25 (10)	C8—C7—H7	121.1
N3—Cu1—N1	87.75 (10)	C6—C7—H7	121.1
N1^i^—Cu1—N1	180.00 (14)	N2—C8—C7	125.3 (3)
N3^i^—Cu1—O1	86.21 (9)	N2—C8—H8	117.3
N3—Cu1—O1	93.79 (9)	C7—C8—H8	117.3
N1^i^—Cu1—O1	91.99 (9)	N2—C9—C10	124.3 (3)
N1—Cu1—O1	88.01 (9)	N2—C9—H9	117.9
N3^i^—Cu1—O1^i^	93.79 (9)	C10—C9—H9	117.9
N3—Cu1—O1^i^	86.21 (9)	C6—C10—C9	119.0 (3)
N1^i^—Cu1—O1^i^	88.01 (9)	C6—C10—H10	120.5
N1—Cu1—O1^i^	91.99 (9)	C9—C10—H10	120.5
O1—Cu1—O1^i^	180.000 (1)	N3—C11—C12	123.8 (3)
C3—S1—C6	103.18 (15)	N3—C11—H11	118.1
C13—S2—C16	101.57 (15)	C12—C11—H11	118.1
N5—O1—Cu1	159.3 (2)	C11—C12—C13	119.2 (3)
C5—N1—C1	116.5 (3)	C11—C12—H12	120.4
C5—N1—Cu1	122.5 (2)	C13—C12—H12	120.4
C1—N1—Cu1	120.4 (2)	C12—C13—C14	117.5 (3)
C8—N2—C9	115.3 (3)	C12—C13—S2	124.7 (3)
C15—N3—C11	116.8 (3)	C14—C13—S2	117.8 (3)
C15—N3—Cu1	121.7 (2)	C15—C14—C13	119.6 (3)
C11—N3—Cu1	121.4 (2)	C15—C14—H14	120.2
C20—N4—C18	115.0 (3)	C13—C14—H14	120.2
O3—N5—O1	122.4 (4)	N3—C15—C14	123.1 (3)
O3—N5—O2	119.9 (4)	N3—C15—H15	118.4
O1—N5—O2	117.7 (3)	C14—C15—H15	118.4
N1—C1—C2	122.9 (3)	C17—C16—C19	117.9 (3)
N1—C1—H1	118.5	C17—C16—S2	121.4 (3)
C2—C1—H1	118.5	C19—C16—S2	120.7 (3)
C1—C2—C3	120.0 (3)	C16—C17—C18	119.1 (4)
C1—C2—H2	120.0	C16—C17—H17	120.4
C3—C2—H2	120.0	C18—C17—H17	120.4
C4—C3—C2	117.2 (3)	N4—C18—C17	124.5 (4)
C4—C3—S1	125.1 (3)	N4—C18—H18	117.7
C2—C3—S1	117.7 (2)	C17—C18—H18	117.7
C5—C4—C3	119.3 (3)	C16—C19—C20	118.4 (4)
C5—C4—H4	120.4	C16—C19—H19	120.8
C3—C4—H4	120.4	C20—C19—H19	120.8
N1—C5—C4	123.9 (3)	N4—C20—C19	125.0 (4)
N1—C5—H5	118.1	N4—C20—H20	117.5
C4—C5—H5	118.1	C19—C20—H20	117.5
C10—C6—C7	118.1 (3)		
			
N3^i^—Cu1—O1—N5	−120.2 (7)	C3—S1—C6—C10	−127.2 (3)
N3—Cu1—O1—N5	59.8 (7)	C3—S1—C6—C7	55.2 (3)
N1^i^—Cu1—O1—N5	−32.6 (7)	C10—C6—C7—C8	2.2 (5)
N1—Cu1—O1—N5	147.4 (7)	S1—C6—C7—C8	179.8 (3)
N3—Cu1—N1—C5	−89.3 (3)	C9—N2—C8—C7	1.1 (6)
O1—Cu1—N1—C5	176.8 (3)	C6—C7—C8—N2	−3.0 (6)
O1^i^—Cu1—N1—C5	−3.2 (3)	C8—N2—C9—C10	1.6 (6)
N3^i^—Cu1—N1—C1	−98.5 (2)	C7—C6—C10—C9	0.2 (5)
N3—Cu1—N1—C1	81.5 (2)	S1—C6—C10—C9	−177.4 (3)
O1—Cu1—N1—C1	−12.3 (2)	N2—C9—C10—C6	−2.3 (6)
O1^i^—Cu1—N1—C1	167.7 (2)	C15—N3—C11—C12	−0.6 (4)
N1^i^—Cu1—N3—C15	57.4 (2)	Cu1—N3—C11—C12	176.5 (2)
N1—Cu1—N3—C15	−122.6 (2)	N3—C11—C12—C13	0.0 (5)
O1—Cu1—N3—C15	−34.7 (2)	C11—C12—C13—C14	0.5 (5)
O1^i^—Cu1—N3—C15	145.3 (2)	C11—C12—C13—S2	178.0 (2)
N1^i^—Cu1—N3—C11	−119.5 (2)	C16—S2—C13—C12	23.8 (3)
N1—Cu1—N3—C11	60.5 (2)	C16—S2—C13—C14	−158.6 (3)
O1—Cu1—N3—C11	148.3 (2)	C12—C13—C14—C15	−0.3 (5)
O1^i^—Cu1—N3—C11	−31.7 (2)	S2—C13—C14—C15	−178.0 (2)
Cu1—O1—N5—O3	166.9 (5)	C11—N3—C15—C14	0.8 (4)
Cu1—O1—N5—O2	−14.5 (9)	Cu1—N3—C15—C14	−176.3 (2)
C5—N1—C1—C2	3.5 (5)	C13—C14—C15—N3	−0.4 (5)
Cu1—N1—C1—C2	−167.9 (2)	C13—S2—C16—C17	−111.6 (3)
N1—C1—C2—C3	0.1 (5)	C13—S2—C16—C19	69.8 (3)
C1—C2—C3—C4	−3.6 (5)	C19—C16—C17—C18	−0.2 (5)
C1—C2—C3—S1	177.4 (2)	S2—C16—C17—C18	−179.0 (3)
C6—S1—C3—C4	16.6 (3)	C20—N4—C18—C17	−0.3 (6)
C6—S1—C3—C2	−164.5 (3)	C16—C17—C18—N4	−0.3 (6)
C2—C3—C4—C5	3.5 (5)	C17—C16—C19—C20	1.2 (5)
S1—C3—C4—C5	−177.6 (3)	S2—C16—C19—C20	179.9 (3)
C1—N1—C5—C4	−3.6 (5)	C18—N4—C20—C19	1.4 (6)
Cu1—N1—C5—C4	167.5 (2)	C16—C19—C20—N4	−1.9 (7)
C3—C4—C5—N1	0.2 (5)		

Symmetry codes: (i) −*x*+1, −*y*+2, −*z*+1.

### Hydrogen-bond geometry (Å, °)

**Table:**

*D*—H···*A*	*D*—H	H···*A*	*D*···*A*	*D*—H···*A*
C11—H11···O1^i^	0.93	2.52	3.063 (4)	118
C5—H5···O2^i^	0.93	2.49	3.419 (4)	174
C5—H5···O1^i^	0.93	2.51	3.193 (4)	130
C14—H14···N4^ii^	0.93	2.47	3.279 (5)	146
C1—H1···O1	0.93	2.27	3.008 (4)	135

Symmetry codes: (i) −*x*+1, −*y*+2, −*z*+1; (ii) *x*+1, *y*, *z*.
